# Couples' changing work patterns in the United Kingdom and the United States during the COVID‐19 pandemic

**DOI:** 10.1111/gwao.12661

**Published:** 2021-03-31

**Authors:** Yue Qian, Yang Hu

**Affiliations:** ^1^ Department of Sociology University of British Columbia Vancouver British Columbia Canada; ^2^ Department of Sociology Lancaster University Lancaster UK

**Keywords:** couple, COVID‐19, cross‐national, gender, human capital, pandemic, work

## Abstract

Going beyond a focus on individual‐level employment outcomes, we investigate couples' changing work patterns in the United Kingdom (UK) and the United States (US) during the COVID‐19 pandemic. Analyzing longitudinal panels of 2186 couples from the Understanding Society COVID‐19 Survey (UK) and 2718 couples from the Current Population Survey (US), we assess whether the pandemic has elevated the importance of human capital vis‐à‐vis traditional gender specialization in shaping couples' work patterns. The UK witnessed a notable increase in sole‐worker families with the better‐educated partner working, irrespective of gender. The impact of the pandemic was similar but weaker in the US. In both countries, couples at the bottom 25% of the prepandemic family income distribution experienced the greatest increase in neither partner working but the least growth in sole‐worker arrangements. Through a couple‐level analysis of changing employment patterns, this study highlights the importance of human capital in shaping couples' paid‐work organization during the pandemic, and it reveals the socioeconomic gradient in such organization.

## 1 INTRODUCTION

Over the past several decades, there has been a long progress toward gender equality in the public sphere across many countries (England et al., 2020; Van Bavel et al., 2018). These progresses are manifested in the improvement and growing recognition of women's human capital, propelled by a gender‐gap reversal in education and a rise in female labor force participation (DiPrete & Buchmann, 2013; England et al., 2020). However, the COVID‐19 pandemic has fundamentally reshuffled the gendered organization of work in many countries. Emerging evidence shows that the pandemic has disproportionately hindered women's and particularly mothers' participation in paid work (e.g., Churchill, 2020; Collins et al., 2021; Dias et al., 2020; Qian & Fuller, 2020). The exacerbation of gender inequality in employment in the wake of COVID‐19 thus instills a looming fear that the pandemic may undo decades of progress for women's equality.

Gendered work arrangements and responses to the pandemic are often devised at a family level, which involve negotiation, strategic coordination, and sometimes compromise without choice between partners (P. E. Becker & Moen, 1999; Killewald & García‐Manglano, 2016). This is particularly the case during the pandemic when many countries have taken the family as a basic unit of crisis governance (Public Health England, 2020). Coresident family members weather the storm and face the consequences of the pandemic together, especially during lockdowns (Biroli et al., 2020; Prime et al., 2020). Existing research on individual‐level outcomes has provided important evidence on pandemic‐related gender inequalities in employment (e.g., Churchill, 2020; Collins et al., 2021; Dias et al., 2020; Qian & Fuller, 2020). However, we still know little about how couples' paid‐work patterns have changed during the pandemic—an important gap we aim to fill in this article.

A couple‐level approach to assessing the impact of the COVID‐19 pandemic on partners' paid‐work organization promises to yield new, important insights into the question of whether and when human capital trumps traditional gender specialization (G. S. Becker, 1991). The relationship between gender specialization and human capital features a central place in scholarship and public debates on gender and work (Bittman et al., 2003; Killewald & Gough, 2013). Against the backdrop of a growing female advantage in educational attainment (DiPrete & Buchmann, 2013), the puzzle remains as to why families still often stick to traditional gender scripts and prioritize men's employment. As the pandemic poses a severe challenge to the economic subsistence of many families, it provides a unique opportunity to examine couples' organization of work: in the face of an unprecedented economic strain, do couples' work patterns change in ways that center on maximizing the economic returns to human capital or reinforcing traditional gendered divisions of paid labor?

This study contributes to a nascent body of cross‐national research that jointly considers gender, work, and social class, by extending it to a pandemic setting (Hook, 2015; Musick et al., 2020). Specifically, our empirical analysis draws on comparative data from two liberal welfare regimes, as defined by Esping‐Andersen (1990): the United Kingdom (the Understanding Society COVID‐19 Survey) and the United States (the Current Population Survey). Exploiting the panel design of these surveys, we use couple‐level fixed‐effects models to chart changes and continuity in couples' work patterns between January–February 2020 (before the COVID‐19 pandemic) and April–May 2020 (during the pandemic). To investigate the interplay between human capital and gender specialization, we examine whether the impact of the pandemic on couples' work patterns differs by the educational pairing of partners. As the ability of and necessity for couples to maximize economic returns to human capital during the pandemic vary with families' socioeconomic positions, we also explore the ways in which changes in couples' work patterns differ by their earning power as reflected in their family income level before the pandemic.

## 2 GENDER, HUMAN CAPITAL, AND HOUSEHOLD SPECIALIZATION IN THE PANDEMIC

In *A Treatise on the Family*, G. S. Becker (1991) conceptualized the family as an economic institution. In this institution, partners are cooperative social actors who pursue a joint goal of maximizing the economic utility of the couple unit (G. S. Becker, 1991). To do so, they strategically coordinate and divide paid and unpaid work to specialize in the type of labor one is “best at” (Killewald & Gough, 2013). According to the economic model of household specialization, the partner with greater earning power usually spends more time on paid work so as to maximize the economic return to their human capital (G. S. Becker, 1991). Human capital is typically defined as individuals' skills and knowledge—a resource that can be used to generate economic returns in the labor market (G. S. Becker, 1993). According to P. E. Becker and Moen (1999), education is a key proxy for human capital and income is a key measure of returns to human capital (see also G. S. Becker, 1993), which we follow in this research.

However, decades of gender research have shown that economic rationality does not always prevail. Rather, the normative conception of gender roles shapes couples' work arrangements in ways that prioritize men's employment over that of women, irrespective of partners' human capital (Bittman et al., 2003; Cha, 2010). Predicated on the stereotypical links between femininity and homemaking/caregiving and between masculinity and breadwinning (Fitzsimons, 2017), the notion of gender specialization posits that persisting patriarchal order, rather than economic rationalization, plays a prominent role in determining couple‐level paid‐work arrangements (Killewald & Gough, 2013).

The two contesting theoretical perspectives—namely human capital versus gender specialization—have attracted sustained scholarly attention (Bittman et al., 2003; Killewald & García‐Manglano, 2016). Particularly, given the rise of women in education and employment over the past decades (DiPrete & Buchmann, 2013; England et al., 2020), existing evidence presents a puzzle: despite an increase in women's human capital, the gendered organization of work still often prioritizes men's paid work over that of women (Fitzsimons, 2017). Some scholars attribute this paradox to a persisting gender wage gap, which means the conversion rate of returns to human capital is lower for women (Lips, 2013). Others suggest that despite the prevalence of dual‐earner families, the male breadwinner norm continues to anchor women in homemaking and caregiving roles and marginalizes the female‐breadwinning model as culturally “deviant” (Blom & Hewitt, 2019).

The COVID‐19 outbreak and subsequent lockdown measures present a unique and potentially fruitful context for re‐examining whether human capital trumps gender specialization in couples' paid‐work arrangements. On the one hand, as the global economy plunges into an abrupt, unprecedented decline, the resultant mass unemployment jeopardizes the economic wellbeing of many families (Cocco et al., 2020; Hu, 2020). Therefore, prioritizing human capital over traditional gender roles may be less of a choice but rather a necessity as couples work to maintain their economic subsistence (Carr & Springer, 2010). On the other hand, however, events such as school closure as well as the lengthened time people spend at home during pandemic lockdowns may increase domestic and care demands, often in a gendered way (Hjálmsdóttir & Bjarnadóttir, 2020). In turn, greater domestic and care demands may reinforce traditional gender specialization and hold women back from paid‐work participation (Collins et al., 2021; Dias et al., 2020).

Early evidence depicts a clear pattern of human capital stratification in paid‐work participation and unemployment during the pandemic. Specifically, low‐skilled service and manual jobs that require lower levels of human capital have been hit hardest by the pandemic (Kochharn, 2020; Qian & Fuller, 2020). In contrast, workers with a greater command of human capital are less likely to cut back on or lose their work, as many of them have jobs that can be performed remotely (Kramer & Kramer, 2020). As a result, plummeting employment rates during the pandemic are largely driven by mass layoffs as opposed to workers quitting their jobs (Dias et al., 2020). This means that prioritizing human capital over gender specialization in couples' paid‐work arrangements may not only be a matter of choice; it may also represent a selection mechanism imposed by the pandemic that favors human capital and eliminates low‐skilled workers from the labor force.

The interplay between human capital and couples' work patterns during the pandemic may vary with families' socioeconomic position. As individual earning power tends to be low in low‐income families (Checchi, 2006), adult members of these families, irrespective of gender, often need all hands on the deck to make ends meet. By contrast, members of affluent families tend to have greater earning power, which means that one partner's income alone may suffice for the family to maintain a decent standard of living (Parpart & Stichter, 2016). In this case, it is financially viable for relatively well‐off families to scale back from a dual‐worker model to a sole‐worker model during the pandemic, in order to strike a balance between maintaining their livelihood, minimizing potential health risks, and responding to increasing domestic and care demands.

We also explore potential cross‐national differences between the UK and the US. The two countries make a worthy pair of comparison due to similarities in their welfare regimes but different national responses to the pandemic. As both the UK and the US are liberal‐regime countries (Esping‐Andersen, 1990), the comparison provides an opportunity to assess whether the pandemic's impact on couples' work patterns are to some extent generalizable across countries of the same welfare regime type. The similar welfare regimes also enable us to explore potential differences resulting from distinct responses to the pandemic between the two countries. While the UK government implemented a strict national lockdown starting from March 23rd, the stay‐at‐home order was implemented in a less comprehensive, less strict, and state‐by‐state manner in the US (Our World in Data, 2020). In the UK, the national lockdown involved a near‐complete closure of service and hospitality industries (e.g., bars, restaurants, and hotels), whereas no similar blanket policy of business closure was implemented in the US. Given the UK's tighter enforcement of lockdown policies, low‐skilled workers in the UK who were grounded in their homes may be more likely to be excluded from the labor force than their US counterparts who were, to a lesser extent, obliged by law to stay home.

Although we do not develop formal hypotheses, the above theoretical considerations direct inquiry into several pertinent questions.

1. How have couples' work patterns changed during the pandemic, compared with before the pandemic?
2. Has the pandemic elevated the importance of human capital vis‐à‐vis gender specialization in shaping couples' work patterns?
3. How do changes in couples' work patterns vary with family socioeconomic position?

## 3 DATA AND METHODS

### 3.1 Data

Our UK data are from the Understanding Society (USOC) COVID‐19 Survey and the preceding Wave 9 of USOC. Initiated in 2009, USOC is a nationally representative, longitudinal household survey (McFall, 2012). During the UK's national lockdown, the USOC COVID‐19 Survey collected data from 17,452 respondents in April and 14,811 respondents in May. The survey also asked about respondents' work and family situations in January and February 2020, which we use to establish a prepandemic baseline. Our US data come from the 2019–2020 Current Population Survey (CPS), obtained from the Integrated Public Use Microdata Series (https://cps.ipums.org/cps/). The CPS is a monthly household survey on the US labor force, with a rotating design: household members are surveyed in four consecutive months, left out of the sample for the following 8 months, and then reinterviewed in the following 4 months. While monthly CPS data do not contain income information, we obtain information on prepandemic family income from the CPS Annual Social and Economic Supplement (ASEC) fielded in March 2019. The panel design of the USOC and the CPS allows us to link data on both members of a couple across time to examine couples' work patterns before and during the pandemic (Flood & Pacas, 2017).

### 3.2 Analytical sample

We first limit our sample to married and cohabiting couples in which both partners' information was available immediately before the pandemic (January and/or February 2020), during the pandemic (April and/or May 2020), and in the earlier wave of the surveys (i.e., USOC Wave 9 and 2019 CPS ASEC). After we construct couple‐level longitudinal data, we restrict our sample to working‐age different‐sex couples in which both partners were aged 25–59 years in 2020.^1^ After eliminating a small number of UK cases with missing values (<10% of the original sample),^2^ our UK sample includes 5835 couple‐months (2186 couples) and our US sample includes 6711 couple‐months (2718 couples). Note that USOC Wave 9 and the 2019 CPS ASEC do not contribute to our couple‐month observations but are used to obtain data on prepandemic family income. Couples' work patterns may be affected by the presence of young children at home. In online supplemental tables, we replicated all analyses by limiting our sample to parents of children aged 0–15, which yielded substantively similar results. We thus present our results from the more inclusive sample, the characteristics of which can be found in Appendix A.

### 3.3 Variables

Our dependent variable is a time‐varying measure of couples' work patterns. To create this variable, we first classify respondents into four employment categories based on their weekly work hours (Killewald & Zhuo, 2019):^3^ (1) no work; (2) 1–19 h (marginally employed); (3) 20–34 h (part‐time); and (4) 35 h or more (full‐time). Next, we compare the male and female partners' employment categories to measure couples' work patterns: neither partner worked (both [1]; denoted as “both no work” in tables and figures), dual worker (both partners in the same non‐zero hour category), male sole worker ([1] for the female partner, [2], [3], or [4] for the male partner), male main worker (both nonzero work hours, but the male partner worked more hours than the female partner), and female main/sole worker (the female partner worked more hours than the male partner; hereafter referred to as “female main worker”). We combined “female main worker” and “female sole worker” because of their small sample sizes. We created a dummy variable for each of the five categories.

Our key predictor is a time indicator distinguishing the periods immediately before the pandemic (February)^4^ and during the pandemic (April–May). Moreover, we consider two time‐invariant moderators. The first moderator measures partners' educational pairing as a proxy for their relative (and absolute) human capital: neither partner is highly educated, highly educated male partner only, highly educated female partner only, and two highly educated partners. Our definition of “highly educated” is context‐specific. It refers to people with at least a bachelor's degree in the US and those with any tertiary degree in the UK. In the US, college graduates are more likely than those with less education to have the option to telework and less likely to become unemployed during the pandemic (Kochharn, 2020). In the UK, a tertiary degree is salient in shaping people's labor market opportunities and outcomes (Belfield et al., 2018). Consistent with prior research showing the gender‐gap reversal in education in the UK and the US (DiPrete & Buchmann, 2013; Van Bavel et al., 2018), we find that in both countries, if only one partner is highly educated, it is more likely to be the female, as opposed to the male, partner (Appendix A). Our second moderator—prepandemic family income level—is derived from the quartiles of family income in each country, based on which we group couples into three categories: bottom 25%, middle 50%, and top 25%.

### 3.4 Analytical strategy

Our couple‐level analysis is characterized by three key features: (1) our unit of analysis is the couple (as opposed to individuals); (2) our variables are measured at the couple level, which account for both partners' attributes; and (3) we run couple‐level fixed effects linear probability regression models predicting each category of couples' work arrangements (for a similar approach, see Musick et al., 2020). A couple‐level fixed effects regression model effectively captures within‐couple change in work patterns by controlling for couples' time‐invariant attributes (both observed and unobserved) that may shape partners' paid‐work participation (Winship & Morgan, 1999).

We fit all models separately by country. Our first set of models include only the time‐varying pandemic indicator. The coefficient for the pandemic indicator shows how couples' work patterns changed between February (prior to the pandemic) and April–May (during the pandemic). Our second set of models include the main effect of the pandemic indicator and its interactions with partners' educational pairings, omitting time‐invariant main effects of educational pairings from the model. Our third set of models include the main effect of the pandemic indicator and its interactions with pre‐pandemic family income levels.^5^ To aid the interpretation of our findings, we graph the predicted probabilities of couples' work patterns.

## 4 RESULTS

**FIGURE 1:**
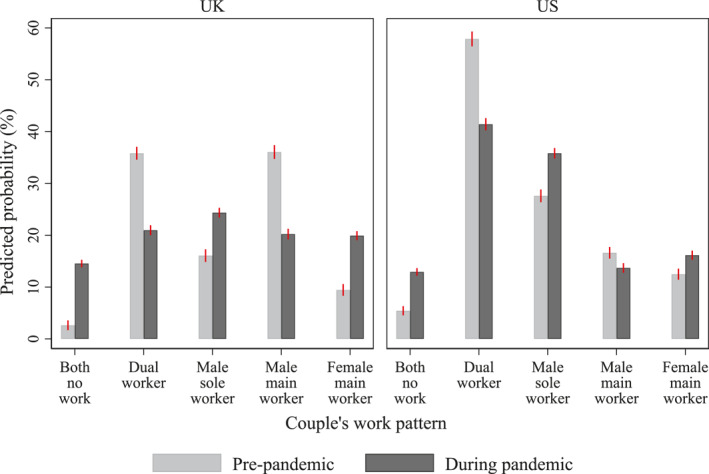
Couples' work patterns before and during the COVID‐19 pandemic (February and April–May), by country. *Note*: Predicted probabilities based on couple fixed‐effects models. Error bars indicate 95% confidence intervals. See Appendix B for full model results

Figure 1 presents couples' work patterns before and during the COVID‐19 pandemic, and full model results are presented in Appendix B. Although the magnitude of changes differed between the UK and the US, similar patterns are observed. For example, both countries witnessed an increase in the arrangement where neither partner worked. While both countries witnessed an increase in the sole‐worker model, the prevalence of dual‐worker and male‐main‐worker models declined. Figure 1 also suggests that changes in couples' work patterns tended to be greater in the UK than in the US. For example, the prevalence of couples in which neither partner worked increased fivefold from 3% to 15% in the UK but increased just over twofold from 5% to 11% in the US. The likelihood of the female‐main‐worker model doubled from 9% to 20% in the UK, but it only changed slightly in the US from 10% to 13%. These cross‐national differences might have resulted from the stricter lockdown measures enforced in the UK than the US.

**FIGURE 2:**
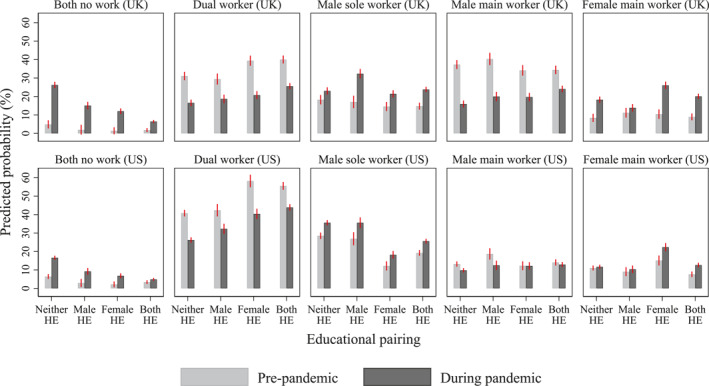
Couples' work patterns before and during the COVID‐19 pandemic (February and April–May), by educational pairing and country. *Note*: Predicted probabilities based on couple fixed‐effects models. Error bars indicate 95% confidence intervals. HE, highly educated. See Appendix C for full model results

How do changes in couples' work patterns vary with partners' educational pairing? Figure 2 presents couples' work patterns by educational pairing and country before and during the pandemic (see Appendix C for full model results). An increase in the prevalence of couples in which neither partner worked was seen across the board, regardless of country and educational pairing, suggesting the widespread negative impact of the pandemic on employment. However, an educational gradient was evident in both countries. The increase in the prevalence of couples in which neither partner worked was most pronounced among couples with two less‐educated partners (from 5% to 26% in the UK and from 6% to 17% in the US), but least prominent among couples with two highly educated partners (from 2% to 6% in the UK and from 3% to 5% in the US).

An increase in the sole‐worker model was evident in both countries, but the increase was uneven across distinct educational pairings. Our findings seem to support the contention that human capital trumped gender, especially in the UK. Specifically, the probability of the male‐sole‐worker model increased the most among couples with a highly educated male partner but a less‐educated female partner, from 17% to 32% (by 15‐percentage‐points) in the UK and from 27% to 36% (by 9‐percentage‐points) in the US. By contrast, among other educational pairings, the probability of the male‐sole‐worker model increased by less than 10‐percentage‐points in the UK and about 6‐percentage‐points in the US. Meanwhile, the probability of the female‐main‐worker model increased the most among couples with a highly educated female partner but a less‐educated male partner, from 10% to 26% (by 16‐percentage‐points) in the UK and from 15% to 22% (by 7‐percentage‐points) in the US, but increased the least among couples with a highly educated male partner only (from 11% to 14% in the UK and from 9% to 10% in the US).

In sum, our results suggest that in both the UK and the US, human capital played a more prominent role than gender specialization in shaping changes in couples' work patterns during the pandemic. The increase in the proportion of couples in which neither partner worked concentrated in the lower end of the human capital spectrum, namely among couples with two less‐educated partners. Meanwhile, the increase in the male‐sole‐worker model was greatest among couples with a better‐educated male partner, whereas the increase in families with women as the sole or primary worker was greatest among couples with a better‐educated female partner. In other words, if two partners differed in educational attainment, human capital seems to have trumped traditional gender roles to shape couples' work patterns during the pandemic.

**FIGURE 3:**
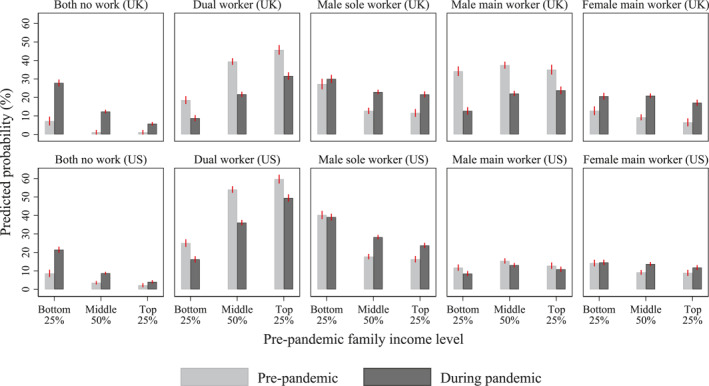
Couples' work patterns before and during the COVID‐19 pandemic (February and April–May), by prepandemic family income level and country. *Note*: Predicted probabilities based on couple fixed‐effects models. Error bars indicate 95% confidence intervals. See Appendix D for full model results

Figure 3 depicts the ways in which the impact of the pandemic on couples' work patterns vary with prepandemic family income level (see Appendix D for full model results). In line with the results for educational pairing, the increase in the prevalence of couples in which neither partner worked was most pronounced among low‐income families. For families in the bottom income quartile, the probability of neither partner working increased from 7% to 28% in the UK and from 9% to 21% in the US. For the middle 50% of families, the probability increased to a smaller degree, from 1% to 12% in the UK and from 4% to 9% in the US. For the top 25% of families, the increase was the smallest, only from 1% to 6% in the UK and from 2% to 4% in the US. These results suggest that the pandemic may have exacerbated pre‐existing socioeconomic inequalities.

Despite an overall increase in the male‐sole‐worker model during the pandemic, the increase was hardly seen in the bottom income quartile and was mainly seen in the 75% of better‐off couples. In the UK, the probability of the male‐sole‐worker model increased only from 27% to 30% in the bottom income quartile, but nearly doubled among the middle 50% of couples (from 13% to 23%) and also in the top income quartile (from 12% to 22%). Similarly, in the US, the probability hardly changed in the bottom quartile (from 40% to 39%), but it increased from 18% to 28% in the middle two quartiles and from 16% to 24% in the top quartile.

The increase in the female‐main‐worker model was also more limited among low‐income families than better‐off families. In the UK, an 8‐percentage‐point increase in the female‐main‐worker model was noted in the bottom income quartile, compared to an increase by 12‐ and 11‐percentage points in the middle and top quartiles, respectively. In the US, the prevalence of the female‐main‐worker model hardly changed among couples at the bottom of the family income distribution, compared to a 4‐percentage‐point increase in the middle two quartiles and a 3‐percentage‐point increase in the top quartile. These results suggest that couples' work patterns during the pandemic was shaped by their socioeconomic position.

## 5 CONCLUSIONS AND DISCUSSION

Recent research focusing on individual‐level outcomes has revealed widening gender inequalities in work hours and employment during the COVID‐19 pandemic (e.g., Churchill, 2020; Collins et al., 2021; Dias et al., 2020; Qian & Fuller, 2020). Extending existing research, this study is the first to offer cross‐national evidence on changes and continuity in couples' work patterns during the pandemic. In so doing, our findings make several important contributions to understanding the impact of the COVID‐19 pandemic on the gendered organization of work and employment.

Adding to the literature on household and feminist economics (G. S. Becker, 1991; Molina, 2011), our findings suggest that human capital rather than gender specialization has come to play a more prominent role in shaping couples' paid‐work patterns in the wake of the pandemic. During the pandemic, we have seen a substantial increase in the proportion of sole‐worker families in which the better‐educated partner, irrespective of gender, participated in paid work. This is especially true in the UK where the lockdown measures were more comprehensively and stringently enforced than in the US. The rise of labor specialization predicated on human capital as opposed to traditional gender roles may be a combined result of couples' proactive attempt to maximize economic returns to their human capital and the protective role of human capital in keeping skilled professionals in tele‐communicable work. While emerging evidence on the gendered impact of COVID‐19 on individuals' employment instills a sense of fear that the pandemic may undo decades of progress toward gender equality (Collins et al., 2021; Qian & Fuller, 2020), our findings ignite a glimmer of hope that the pandemic may have created a condition in which human capital trumps traditional gender roles and thus a catalyst for achieving greater gender equality.

The impact of the pandemic on couples' work patterns varies across the socioeconomic strata. We find that compared to their more affluent and better‐educated counterparts, couples at the bottom of the (prepandemic) family income distribution and couples in which neither partner was highly educated experienced a more dramatic increase in the situation where neither partner worked. This is especially true in the UK, perhaps due to the country's more stringent lockdown and business closure policies than the US. This result suggests that the pandemic has exacerbated pre‐existing socioeconomic inequalities. It remains to be seen whether couples in the bottom socioeconomic stratum were only temporarily out of work by tracing their long‐term trajectory of economic recovery. To aid equitable postpandemic recovery, it would be crucial for governments and employers to attend to the employment needs of low‐income and less‐educated couples. We also found that the increase of sole‐earner couples during the pandemic was much greater among affluent than low‐income families. In fact, there was hardly any increase in the prevalence of sole‐worker families in the bottom income quartile. As a result, low‐income couples were less likely to have moved toward greater gender specialization in paid work during the pandemic. This could be because low‐income couples tended to command lower levels of individual human capital and earning power, which meant that both partners needed to participate in paid work, whenever they could, in order to make ends meet.

Given the quantitative nature of our study, we were unable to identify the specific mechanisms underpinning couples' changing work patterns. The observed changes may be a combined result of couples' negotiation or strategic coordination to maximize family well‐being and couples' compromise without choice in light of mass layoffs amid business shutdowns and organizational downsizing. Further qualitative research is needed to more fully unpack the nuanced explanations for couples' changing work patterns during COVID‐19. Meanwhile, given our focus on couples' paid‐work patterns, the question remains as to whether and how changing work arrangements have evolved in tandem with couples' divisions of household and care work. Future data collection and analysis could usefully examine the work–family interface. Such a focus will help ascertain whether the rising importance of human capital in couples' employment arrangements during the pandemic has led to greater gender equality in the division of unpaid labor or an intensified work–family double‐bind for educated professional women who remained in work. Moreover, with a focus on the immediate impact of the pandemic, we are not able to determine whether the observed changes may persist or cascade into further changes in the long run.

We have made a first attempt to conduct a comparative study of the pandemic's impact on couples' work patterns, although we are unable to systematically explain differences and similarities between the UK and the US. First, we find that the patterns of change in couples' work patterns were similar between the two countries, suggesting the diffuse impact of the COVID‐19 pandemic beyond the confine of specific nation‐states. As both the UK and the US are liberal‐regime states, our observed similarities between the two countries suggest that the pandemic's impact on couples' paid‐work patterns may apply to a broader range of countries adopting a liberal welfare regime (Esping‐Andersen, 1990). Future research could usefully expand our scope to examine the interplay between human capital and gender specialization in reshaping couples' work patterns in other types of welfare regimes (e.g., conservative regimes, social democratic regimes). Second, the magnitude of change in couples' work patterns appeared to be greater in the UK than in the US, suggesting that governmental responses to COVID‐19 may have shaped couple‐level adaptions, coping strategies, and consequences. As social scientists are collecting comparable data across a wide range of countries adopting diverse pandemic mitigation measures, it would be fruitful to more systemically explain cross‐national similarities and differences in the pandemic's impacts on couples' work patterns, when such data become available.

While most research so far has focused exclusively on the labor market implications of the COVID‐19 pandemic for individual workers (e.g., Churchill, 2020; Collins et al., 2021; Dias et al., 2020; Qian & Fuller, 2020), we have taken a couple‐level approach to advance our understanding of the social and economic impacts of the pandemic. Focusing on couples' work patterns, this study acknowledges that gendered patterns of paid work are constructed jointly between partners (Killewald & García‐Manglano, 2016; Musick et al., 2020). Our couple‐level analysis underlines the fact that people often weather and coordinate their responses to the pandemic as a family. Therefore, it is crucial to understand the impact of the pandemic on family collectives rather than individuals.

## Supporting information

Supplementary Material

## Data Availability

The UK data were made available through the UK Data Archive. The US data were made available through the Integrated Public Use Microdata Series (https://cps.ipums.org/cps/). The Current Population Survey is conducted jointly by the US Census Bureau and the US Bureau of Labor Statistics.

## ENDNOTES

^1^
The average age for our analytical sample is 44 years for women and 46 years for men in the UK, and the respective figures are 42 and 44 in the US.

^2^
Little's missing completely at random (MCAR) test showed that the missing values were MCAR (Li, 2013).

^3^
In USOC, respondents were asked to report their average weekly work hours in January–February, April, and May 2020, respectively. The CPS asked respondents to report their work hours during the previous week.

^4^
Hereafter, we use “February” to refer to January–February for the UK sample.

^5^
Due to small cell size, we were not able to examine the three‐way interactions between time, educational pairing, and family income level.

## APPENDIX A Sample characteristics

### A.1

**Table:**

Variable	UK (%)	US (%)
*Couple‐month level*		
April–May (during the pandemic)	62.5	59.5
Couple's work patterns before the pandemic		
Both no work	2.7	4.7
Dual worker	35.4	48.1
Male sole worker	16.3	23.0
Male main worker	35.8	13.9
Female main worker	9.8	10.4
Couples' work patterns during the pandemic		
Both no work	14.5	10.6
Dual worker	21.2	34.6
Male sole worker	24.2	29.9
Male main worker	20.4	11.4
Female main worker	19.7	13.5
*Couple level*		
Educational pairing		
Neither partner highly educated	28.2	44.4
Male highly educated only	14.7	9.0
Female highly educated only	21.8	14.8
Both partners highly educated	35.3	31.8
Pre‐pandemic family income level		
Bottom 25%	25.0	24.9
Middle 50%	50.0	50.1
Top 50%	25.0	25.0
*N* (couple–month)	5,835	6,711
*N* (couple)	2,186	2,718

*Note*: Column percentages may not add up to 100% due to rounding. Unweighted statistics.

## APPENDIX B Couple fixed‐effects models predicting couples' work patterns, by country

### B.1

**Table:**

Predictors	UK	US	UK–US difference (*p*)
*Both no work*			
April–May (ref. = February)	0.12***	0.06***	<0.001
	(0.01)	(0.00)	
Constant	0.03***	0.05***	
	(0.00)	(0.00)	
Adjusted *R* ^*2*^	0.66	0.70	
*Dual worker*			
April–May (ref. = February)	−0.15***	−0.14***	ns
	(0.01)	(0.01)	
Constant	0.36***	0.48***	
	(0.01)	(0.01)	
Adjusted *R* ^2^	0.73	0.76	
*Male sole worker*			
April–May (ref. = February)	0.08***	0.07***	ns
	(0.01)	(0.01)	
Constant	0.16***	0.23***	
	(0.01)	(0.01)	
Adjusted *R* ^2^	0.69	0.78	
*Male main worker*			
April–May (ref. = February)	−0.16***	−0.02***	<0.001
	(0.01)	(0.01)	
Constant	0.36***	0.14***	
	(0.01)	(0.00)	
Adjusted *R* ^2^	0.68	0.67	
*Female main worker*			
April–May (ref. = February)	0.10***	0.03***	<0.001
	(0.01)	(0.01)	
Constant	0.09***	0.10***	
	(0.01)	(0.00)	
Adjusted *R* ^2^	0.67	0.70	
*N* (couple–month)	5,835	6,711	
*N* (couple)	2,186	2,718	

*Note*: ns is not statistically significant at the 10% level. Standard errors in parentheses. Linear probability models. Underlying models for Figure 1.

Abbreviation: ref., reference category.

****p* < 0.001 (two‐tailed tests).

## APPENDIX C Couple fixed‐effects models predicting couples' work patterns, by educational pairing and country

### C.1

**Table:**

	Neither HE		UK–US diff. (*p*)	Male HE		UK–US diff. (*p*)	Female HE		UK–US diff. (*p*)	Both HE		UK–US diff. (*p*)
Predictors	UK	US		UK	US		UK	US		UK	US	
*Both no work*												
April–May (ref. = February)	0.21***	0.10***	<0.001	0.13***	0.06***	<0.01	0.11***	0.05***	<0.001	0.05***	0.01*	<0.001
	(0.01)	(0.01)		(0.02)	(0.01)		(0.01)	(0.01)		(0.01)	(0.01)	
Constant	0.05***	0.06***		0.02	0.03**		0.01	0.02**		0.02**	0.03***	
	(0.01)	(0.01)		(0.01)	(0.01)		(0.01)	(0.01)		(0.01)	(0.01)	
Adjusted *R* ^2^	0.68	0.71		0.61	0.72		0.62	0.68		0.69	0.69	
*Dual worker*												
April–May (ref. = February)	−0.15***	−0.15***	ns	−0.11***	−0.10***	ns	−0.19***	−0.18***	ns	−0.14***	−0.12***	ns
	(0.01)	(0.01)		(0.02)	(0.02)		(0.02)	(0.02)		(0.01)	(0.01)	
Constant	0.31***	0.41***		0.29***	0.42***		0.39***	0.58***		0.40***	0.56***	
	(0.01)	(0.01)		(0.02)	(0.02)		(0.01)	(0.02)		(0.01)	(0.01)	
Adjusted *R* ^2^	0.71	0.73		0.74	0.82		0.72	0.72		0.74	0.77	
*Male sole worker*												
April–May (ref. = February)	0.05**	0.07***	ns	0.15***	0.09***	<0.05	0.07***	0.06***	ns	0.09***	0.06***	<0.10
	(0.02)	(0.01)		(0.02)	(0.02)		(0.02)	(0.02)		(0.01)	(0.01)	
Constant	0.18***	0.29***		0.17***	0.27***		0.15***	0.12***		0.15***	0.19***	
	(0.01)	(0.01)		(0.02)	(0.02)		(0.01)	(0.01)		(0.01)	(0.01)	
Adjusted *R* ^2^	0.62	0.76		0.70	0.79		0.70	0.72		0.74	0.82	
*Male main worker*												
April–May (ref. = February)	−0.21***	−0.03***	<0.001	−0.20***	−0.06**	<0.001	−0.14***	−0.00	<0.001	−0.10***	−0.01	<0.001
	(0.02)	(0.01)		(0.02)	(0.02)		(0.02)	(0.02)		(0.01)	(0.01)	
Constant	0.37***	0.13***		0.40***	0.19***		0.34***	0.12***		0.34***	0.14***	
	(0.01)	(0.01)		(0.02)	(0.02)		(0.01)	(0.01)		(0.01)	(0.01)	
Adjusted *R* ^2^	0.68	0.63		0.72	0.73		0.68	0.68		0.68	0.69	
*Female main worker*												
April–May (ref. = February)	0.10***	0.01	<0.001	0.03	0.01	ns	0.16***	0.07***	<0.001	0.11***	0.05***	<0.001
	(0.01)	(0.01)		(0.02)	(0.02)		(0.02)	(0.02)		(0.01)	(0.01)	
Constant	0.08***	0.11***		0.11***	0.09***		0.10***	0.15***		0.09***	0.08***	
	(0.01)	(0.01)		(0.01)	(0.01)		(0.01)	(0.01)		(0.01)	(0.01)	
Adjusted *R* ^2^	0.62	0.68		0.68	0.72		0.69	0.75		0.69	0.69	
*N* (couple–month)	1652	2972		869	599		1253	989		2061	2151	
*N* (couple)	616	1208		321	244		477	402		772	864	

*Note*: ns is not statistically significant at the 10% level. Standard errors in parentheses. Linear probability models. Underlying models for Figure 2.

Abbreviations: diff., difference; HE, highly educated; ref., reference category.

**p* < 0.05, ***p* < 0.01, ****p* < 0.001 (two‐tailed tests).

## APPENDIX D Couple fixed‐effects models predicting couples' work patterns, by prepandemic family income level and country

### D.1

**Table:**

	Bottom 25%		UK–US	Middle 50%		UK–US	Top 25%		UK–US
Predictors	UK	US	difference (*p*)	UK	US	difference (*p*)	UK	US	difference (*p*)
*Both no work*									
April–May (ref. = February)	0.21***	0.13***	<0.001	0.11***	0.05***	<0.001	0.05***	0.02*	<0.01
	(0.02)	(0.01)		(0.01)	(0.01)		(0.01)	(0.01)	
Constant	0.07***	0.09***		0.01	0.04***		0.01	0.02***	
	(0.01)	(0.01)		(0.01)	(0.00)		(0.01)	(0.01)	
Adjusted *R* ^2^	0.69	0.71		0.61	0.71		0.65	0.61	
*Dual worker*									
April–May (ref. = February)	−0.10***	−0.09***	ns	−0.18***	−0.18***	ns	−0.14***	−0.10***	<0.10
	(0.01)	(0.01)		(0.01)	(0.01)		(0.02)	(0.02)	
Constant	0.19***	0.25***		0.39***	0.54***		0.46***	0.60***	
	(0.01)	(0.01)		(0.01)	(0.01)		(0.01)	(0.01)	
Adjusted *R* ^2^	0.64	0.72		0.72	0.73		0.75	0.77	
*Male sole worker*									
April–May (ref. = February)	0.03	−0.01	<0.10	0.10***	0.11***	ns	0.10***	0.07***	ns
	(0.02)	(0.01)		(0.01)	(0.01)		(0.01)	(0.01)	
Constant	0.27***	0.40***		0.13***	0.18***		0.12***	0.16***	
	(0.01)	(0.01)		(0.01)	(0.01)		(0.01)	(0.01)	
Adjusted *R* ^2^	0.66	0.78		0.69	0.76		0.73	0.81	
*Male main worker*									
April–May (ref. = February)	−0.21***	−0.03**	<0.001	−0.15***	−0.02*	<0.001	−0.11***	−0.02	<0.001
	(0.02)	(0.01)		(0.01)	(0.01)		(0.02)	(0.01)	
Constant	0.34***	0.12***		0.37***	0.15***		0.35***	0.13***	
	(0.01)	(0.01)		(0.01)	(0.01)		(0.01)	(0.01)	
Adjusted *R* ^2^	0.65	0.66		0.69	0.65		0.64	0.70	
*Female main worker*									
April–May (ref. = February)	0.08***	0.00	<0.001	0.12***	0.04***	<0.001	0.11***	0.03**	<0.001
	(0.02)	(0.01)		(0.01)	(0.01)		(0.01)	(0.01)	
Constant	0.13***	0.14***		0.09***	0.09***		0.07***	0.09***	
	(0.01)	(0.01)		(0.01)	(0.01)		(0.01)	(0.01)	
Adjusted *R* ^2^	0.67	0.73		0.67	0.66		0.64	0.71	
*N* (couple–month)	1443	1675		2933	3355		1459	1681	
*N* (couple)	547	677		1093	1361		546	680	

*Note*: Standard errors in parentheses. ns is not statistically significant at the 10% level. Linear probability models. Underlying models for Figure 3.

Abbreviation: ref., reference category.

**p* < 0.05, ***p* < 0.01, ****p* < 0.001 (two‐tailed tests).
