# Supplementary material for: Couples' changing work patterns in the United Kingdom and the United States during the COVID‐19 pandemic
Source: Gend Work Organ. 2021 Mar 31;28(Suppl 2):535–53. doi: 10.1111/gwao.12661 (PMC8250666; doi:10.1111/gwao.12661)
Supplement: Supplementary file 1 — Supplementary Material [file GWAO-28-535-s001.docx]

**Supplemental Analysis**

**for**

**Couples’ Changing Work Patterns**

**in the United Kingdom and the United States during the COVID-19 Pandemic**

**(Limiting sample to parents with children age 0–15 in household)**

| **SUPPLEMENTAL TABLE S1.** Couple fixed-effects models predicting couples’ work patterns, by country | | | |
| --- | --- | --- | --- |
| Predictors | UK | US | UK–US difference (*p*) |
| *Both no work* |  |  |  |
| April–May (ref. = February) | 0.11*** | 0.07*** | < 0.001 |
|  | (0.01) | (0.01) |  |
| Constant | 0.02* | 0.04*** |  |
|  | (0.01) | (0.01) |  |
| Adjusted *R^2^* | 0.62 | 0.67 |  |
| *Dual worker* |  |  |  |
| April–May (ref. = February) | –0.12*** | –0.14*** | ns |
|  | (0.01) | (0.01) |  |
| Constant | 0.30*** | 0.45*** |  |
|  | (0.01) | (0.01) |  |
| Adjusted *R^2^* | 0.73 | 0.76 |  |
| *Male sole worker* |  |  |  |
| April–May (ref. = February) | 0.10*** | 0.07*** | < 0.10 |
|  | (0.01) | (0.01) |  |
| Constant | 0.19*** | 0.27*** |  |
|  | (0.01) | (0.01) |  |
| Adjusted *R^2^* | 0.69 | 0.79 |  |
| *Male main worker* |  |  |  |
| April–May (ref. = February) | –0.19*** | –0.03*** | < 0.001 |
|  | (0.01) | (0.01) |  |
| Constant | 0.42*** | 0.16*** |  |
|  | (0.01) | (0.01) |  |
| Adjusted *R^2^* | 0.68 | 0.67 |  |
| *Female main worker* |  |  |  |
| April–May (ref. = February) | 0.10*** | 0.04*** | < 0.001 |
|  | (0.01) | (0.01) |  |
| Constant | 0.07*** | 0.08*** |  |
|  | (0.01) | (0.01) |  |
| Adjusted *R^2^* | 0.67 | 0.70 |  |
| *N* (couple–month) | 3,199 | 3,600 |  |
| *N* (couple) | 1,218 | 1,460 |  |
| *Note*: Ref. = Reference category. ns = not statistically significant at the 10% level. Standard errors in parentheses. Linear probability models.  * *p* < 0.05, *** *p* < 0.001 (two-tailed tests). | | | |

| **SUPPLEMENTAL TABLE S2.** Couple fixed-effects models predicting couples’ work patterns, by educational pairing and country | | | | | | | | | | | | |
| --- | --- | --- | --- | --- | --- | --- | --- | --- | --- | --- | --- | --- |
|  | Neither HE | | UK–US diff. (*p*) | Male HE | | UK–US diff. (*p*) | Female HE | | UK–US diff. (*p*) | Both HE | | UK–US diff. (*p*) |
| Predictors | UK | US |  | UK | US |  | UK | US |  | UK | US |  |
| *Both no work* |  |  |  |  |  |  |  |  |  |  |  |  |
| April–May (ref. = Feb.) | 0.22*** | 0.12*** | < 0.001 | 0.11*** | 0.06** | ns | 0.10*** | 0.05*** | < 0.05 | 0.05*** | 0.02 | < 0.05 |
|  | (0.02) | (0.01) |  | (0.02) | (0.02) |  | (0.02) | (0.01) |  | (0.01) | (0.01) |  |
| Constant | 0.03* | 0.05*** |  | 0.01 | 0.03 |  | 0.01 | 0.01 |  | 0.01 | 0.03*** |  |
|  | (0.02) | (0.01) |  | (0.02) | (0.02) |  | (0.01) | (0.01) |  | (0.01) | (0.01) |  |
| Adjusted *R^2^* | 0.64 | 0.71 |  | 0.57 | 0.72 |  | 0.63 | 0.68 |  | 0.60 | 0.69 |  |
| *Dual worker* |  |  |  |  |  |  |  |  |  |  |  |  |
| April–May (ref. = Feb.) | –0.10*** | –0.14*** | ns | –0.07** | –0.11** | ns | –0.17*** | –0.21*** | ns | –0.14*** | –0.13*** | ns |
|  | (0.02) | (0.02) |  | (0.03) | (0.03) |  | (0.02) | (0.03) |  | (0.02) | (0.02) |  |
| Constant | 0.24*** | 0.35*** |  | 0.22*** | 0.36*** |  | 0.33*** | 0.58*** |  | 0.36*** | 0.53*** |  |
|  | (0.01) | (0.01) |  | (0.02) | (0.03) |  | (0.02) | (0.02) |  | (0.01) | (0.01) |  |
| Adjusted *R^2^* | 0.74 | 0.73 |  | 0.69 | 0.82 |  | 0.72 | 0.72 |  | 0.74 | 0.77 |  |
| *Male sole worker* |  |  |  |  |  |  |  |  |  |  |  |  |
| April–May (ref. = Feb.) | 0.03 | 0.06*** | ns | 0.19*** | 0.12*** | ns | 0.09*** | 0.04 | ns | 0.11*** | 0.08*** | ns |
|  | (0.03) | (0.02) |  | (0.03) | (0.03) |  | (0.02) | (0.02) |  | (0.02) | (0.01) |  |
| Constant | 0.25*** | 0.35*** |  | 0.19*** | 0.30*** |  | 0.18*** | 0.14*** |  | 0.14*** | 0.22*** |  |
|  | (0.02) | (0.01) |  | (0.02) | (0.03) |  | (0.02) | (0.02) |  | (0.01) | (0.01) |  |
| Adjusted *R^2^* | 0.64 | 0.76 |  | 0.70 | 0.79 |  | 0.71 | 0.72 |  | 0.71 | 0.82 |  |
| *Male main worker* |  |  |  |  |  |  |  |  |  |  |  |  |
| April–May (ref. = Feb.) | –0.23*** | –0.05*** | < 0.001 | –0.28*** | –0.10** | < 0.001 | –0.17*** | 0.01 | < 0.001 | –0.13*** | –0.01 | < 0.001 |
|  | (0.02) | (0.01) |  | (0.03) | (0.03) |  | (0.03) | (0.03) |  | (0.02) | (0.01) |  |
| Constant | 0.40*** | 0.15*** |  | 0.49*** | 0.28*** |  | 0.40*** | 0.17*** |  | 0.43*** | 0.16*** |  |
|  | (0.02) | (0.01) |  | (0.02) | (0.02) |  | (0.02) | (0.02) |  | (0.02) | (0.01) |  |
| Adjusted *R^2^* | 0.70 | 0.63 |  | 0.72 | 0.73 |  | 0.67 | 0.68 |  | 0.67 | 0.69 |  |
| *Female main worker* |  |  |  |  |  |  |  |  |  |  |  |  |
| April–May (ref. = Feb.) | 0.08*** | 0.00 | < 0.001 | 0.06** | 0.03 | ns | 0.15*** | 0.11*** | ns | 0.10*** | 0.05*** | < 0.01 |
|  | (0.02) | (0.01) |  | (0.02) | (0.02) |  | (0.02) | (0.02) |  | (0.02) | (0.01) |  |
| Constant | 0.08*** | 0.10*** |  | 0.09*** | 0.04** |  | 0.08*** | 0.10*** |  | 0.06*** | 0.06*** |  |
|  | (0.01) | (0.01) |  | (0.02) | (0.01) |  | (0.02) | (0.02) |  | (0.01) | (0.01) |  |
| Adjusted *R^2^* | 0.63 | 0.68 |  | 0.72 | 0.72 |  | 0.72 | 0.75 |  | 0.64 | 0.69 |  |
| *N* (couple–month) | 796 | 1,489 |  | 472 | 281 |  | 724 | 508 |  | 1,207 | 1,322 |  |
| *N* (couple) | 304 | 606 |  | 177 | 114 |  | 280 | 204 |  | 457 | 536 |  |
| *Note*: HE = Highly educated. Ref. = Reference category. Feb. = February. Diff. = Difference. ns = not statistically significant at the 10% level. Standard errors in parentheses. Linear probability models.  * *p* < 0.05, ** *p* < 0.01, *** *p* < 0.001 (two-tailed tests). | | | | | | | | | | | | |

| **SUPPLEMENTAL TABLE S3.** Couple fixed-effects models predicting couples’ work patterns, by pre-pandemic family income level and country | | | | | | | | | |
| --- | --- | --- | --- | --- | --- | --- | --- | --- | --- |
|  | Bottom 25% | | UK–US  difference (*p*) | Middle 50% | | UK–US  difference (*p*) | Top 25% | | UK–US  difference (*p*) |
| Predictors | UK | US |  | UK | US |  | UK | US |  |
| *Both no work* |  |  |  |  |  |  |  |  |  |
| April–May (ref. = Feb.) | 0.22*** | 0.14*** | < 0.01 | 0.09*** | 0.05*** | < 0.01 | 0.03** | 0.03* | < 0.01 |
|  | (0.02) | (0.02) |  | (0.01) | (0.01) |  | (0.01) | (0.01) |  |
| Constant | 0.04* | 0.06*** |  | 0.01 | 0.03*** |  | 0.01 | 0.02* |  |
|  | (0.02) | (0.01) |  | (0.01) | (0.01) |  | (0.01) | (0.01) |  |
| Adjusted *R^2^* | 0.64 | 0.69 |  | 0.62 | 0.67 |  | 0.55 | 0.54 |  |
| *Dual worker* |  |  |  |  |  |  |  |  |  |
| April–May (ref. = Feb.) | –0.07*** | –0.08*** | ns | –0.15*** | –0.18*** | ns | –0.13*** | –0.13*** | ns |
|  | (0.02) | (0.02) |  | (0.01) | (0.02) |  | (0.02) | (0.02) |  |
| Constant | 0.15*** | 0.23*** |  | 0.32*** | 0.51*** |  | 0.43*** | 0.58*** |  |
|  | (0.01) | (0.01) |  | (0.01) | (0.01) |  | (0.02) | (0.02) |  |
| Adjusted *R^2^* | 0.71 | 0.75 |  | 0.72 | 0.75 |  | 0.72 | 0.76 |  |
| *Male sole worker* |  |  |  |  |  |  |  |  |  |
| April–May (ref. = Feb.) | 0.03 | –0.01 | ns | 0.13*** | 0.11*** | ns | 0.11*** | 0.08*** | ns |
|  | (0.03) | (0.02) |  | (0.02) | (0.01) |  | (0.02) | (0.02) |  |
| Constant | 0.31*** | 0.45*** |  | 0.15*** | 0.20*** |  | 0.12*** | 0.19*** |  |
|  | (0.02) | (0.01) |  | (0.01) | (0.01) |  | (0.02) | (0.01) |  |
| Adjusted *R^2^* | 0.64 | 0.80 |  | 0.70 | 0.76 |  | 0.72 | 0.80 |  |
| *Male main worker* |  |  |  |  |  |  |  |  |  |
| April–May (ref. = Feb.) | –0.26*** | –0.05** | < 0.001 | –0.18*** | –0.03 | < 0.001 | –0.12*** | –0.02 | < 0.001 |
|  | (0.02) | (0.02) |  | (0.02) | (0.01) |  | (0.02) | (0.02) |  |
| Constant | 0.39*** | 0.14*** |  | 0.45*** | 0.19*** |  | 0.41*** | 0.14*** |  |
|  | (0.02) | (0.01) |  | (0.01) | (0.01) |  | (0.02) | (0.01) |  |
| Adjusted *R^2^* | 0.65 | 0.67 |  | 0.68 | 0.65 |  | 0.71 | 0.71 |  |
| *Female main worker* |  |  |  |  |  |  |  |  |  |
| April–May (ref. = Feb.) | 0.08*** | 0.01 | < 0.01 | 0.11*** | 0.05*** | < 0.001 | 0.11*** | 0.05*** | < 0.05 |
|  | (0.02) | (0.01) |  | (0.01) | (0.01) |  | (0.02) | (0.01) |  |
| Constant | 0.11*** | 0.12*** |  | 0.07*** | 0.07*** |  | 0.04* | 0.07*** |  |
|  | (0.01) | (0.01) |  | (0.01) | (0.01) |  | (0.01) | (0.01) |  |
| Adjusted *R^2^* | 0.69 | 0.75 |  | 0.68 | 0.65 |  | 0.60 | 0.72 |  |
| *N* (couple–month) | 878 | 964 |  | 1,581 | 1,756 |  | 740 | 880 |  |
| *N* (couple) | 341 | 390 |  | 595 | 715 |  | 282 | 355 |  |
| *Note*: Ref. = Reference category. Feb. = February. Standard errors in parentheses. ns = not statistically significant at the 10% level. Linear probability models.  * *p* < 0.05, ** *p* < 0.01, *** *p* < 0.001 (two-tailed tests). | | | | | | | | | |
